# Recommendations for the surveillance of multidrug-resistant bacteria in Italian long-term care facilities by the GLISTer working group of the Italian Association of Clinical Microbiologists (AMCLI)

**DOI:** 10.1186/s13756-020-00771-0

**Published:** 2020-07-13

**Authors:** Richard Aschbacher, Leonardo Pagani, Roberta Migliavacca, Laura Pagani, the GLISTer (Gruppo di Lavoro per lo Studio delle Infezioni nelle Residenze Sanitarie Assistite e Strutture Assimilabili) working group, Massimo Confalonieri, Claudio Farina, Paolo Fazii, Francesco Luzzaro, Roberto Rigoli, Melissa Spalla

**Affiliations:** 1 Microbiology and Virology Laboratory, Bolzano Central Hospital, Bolzano, Italy; 2 Infectious Diseases Unit, Bolzano Central Hospital, Bolzano, Italy; 3 Department of Clinical-Surgical, Diagnostic and Pediatric Sciences, Unit of Microbiology and Clinical Microbiology, University of Pavia, Pavia, Italy

**Keywords:** Italy, LTCF, MDRO, Surveillance, Screening, Infection control, Prevention

## Abstract

Long-term care facilities (LTCFs) are an important reservoir of multidrug-resistant organisms (MDROs). Colonization of LTCF residents by MDROs is generally higher in Italy compared to other European countries. The present review by the working group for the study of infections in LTCFs (GLISTer) of the Italian Association of Clinical Microbiologists (AMCLI) aims to propose criteria for a laboratory-based surveillance of MDROs in Italian LTCFs.

We recommend the adhesion to three levels of laboratory-based MDROs surveillance in LTCFs: i) mandatory MDRO surveillance by cumulative retrospective analysis of antimicrobial susceptibility data, obtained as part of routine care of clinical specimens. ii) strongly recommended surveillance by active rectal swab cultures or molecular screening to determine colonization with carbapenemase-producing *Enterobacterales*, should a resident be proven infected. iii) voluntary surveillance by prospective MDRO surveys, mainly based on point prevalence colonization studies, allowing to determine the MDROs baseline prevalence in the facility.

Laboratory-based surveillance of MDROs in LTCFs is aimed at providing useful epidemiological information to healthcare providers operating in the facility, but it is only effective if the collected data are used for infection prevention and control purposes, targeting the peculiar aspects of LTCFs.

## Introduction

Long-term care facilities (LTCFs) are institutions that provide skilled nursing care to residents in need of assistance with activities of daily living. LTCFs encompass nursing homes, residential care centers, chronic disease hospitals, rehabilitation centers and institutions for mentally handicapped persons [1]. These facilities are functionally the home for residents, usually elderly and in declining health status, often staying for months to years. Due to the ageing population in Italy (present life expectancy 80.1 years for males and 84.7 years for females) LTCFs play an important role in the Italian healthcare system; in December 2013, 12.200 LTCFs were counted, hosting 384.000 people, and these figures are intended to increase during the next decades [http://www.istat.it/].

The frequently older, sicker residents cared for in LTCFs, have a variety of risk factors for colonization and infection by multidrug-resistant organisms (MDROs). Pathogen cross-transmission within LTCFs is a significant issue and residents of these facilities are frequently moved between the acute and the long-term care settings [2]. For these reasons, LTCFs are an important reservoir for MDROs, such as methicillin-resistant *Staphylococcus aureus* (MRSA), vancomycin-resistant enterococci (VRE), *Pseudomonas aeruginosa* or *Acinetobacter baumannii* with MDR phenotypes (expressing resistance to ≥3 classes of antibiotics) and *Enterobacterales* producing extended-spectrum β-lactamases (ESBLs), high-level cephalosporinases (AmpCs) and/or carbapenemases [3, 4]; these MDROs have been included in the present surveillance recommendations.

Surveillance of MDROs in a healthcare institution is of pivotal importance for guiding prevention, therapy and control of infectious diseases caused by these organisms [5]. The urgent need for MDRO surveillance recommendations in Italian LTCFs has been highlighted during the National Congress of AMCLI in Rimini, 2017 [6], and by the national plan for contrasting antimicrobial resistance 2017–20, approved by the Italian Presidency of the Council of Ministers (PNCAR) [7]. Therefore, the Italian working group for the study of infections in LTCFs (GLISTer) of the Association of Italian Clinical Microbiologists (AMCLI), has been prompted to define and recommend general indications for the surveillance of MDROs in Italian LTCFs [8]. For literature search we adopted a search strategy in the Medline/Pubmed database including the following search terms: (nursing home* OR long term care facility*) AND (colonization OR multi drug resistan* OR ESBL OR MRSA OR VRE OR carbapenemase*) AND Italy. We restricted the search to the dates range 01.01.2000–30.08.2019 but did not impose any language restriction. Applying this search strategy, we identified 30 articles; from the reference lists of the above articles we retrieved further studies. Moreover, we included a Poster Abstract from the National Congress AMCLI 2017, the “Piano Nazionale per il Contrasto dell’antimicrobico-resistenza 2017-20 (PNCAR)”, Clinical and Laboratory Standards Institute (CLSI) guidelines for analysis and presentation of cumulative antimicrobial susceptibility test data, and international and national guidelines for infection prevention and control in LTCFs. At least two authors screened every abstract and full-text article or guideline, the results have been collegially discussed before the inclusion in the review article.

The recommendations are intended to be used by local and regional bodies, comprising LTCF physicians, infectious disease specialists and nurses, infection preventionists, LTCF administrations, referral hospital physicians, clinical microbiologists and administrations.

## MDR bacteria in Italian LTCFs

Dwelling in a LTCF is an independent risk factor for bloodstream infections by MDROs [9], and Italy has one of the highest prevalence of MDROs isolated from blood cultures in European countries, especially for MRSA, carbapenemase-producing *A. baumannii* and ESBL- or carbapenemase-producing *Enterobacterales* (CPE) [10]. A systematic review of studies on colonization by MDROs from Italian LTCFs, on risk factors for colonization and on molecular characteristics of clinical isolates also in comparison with other European countries, has been published recently [11]. Italian review data show an MRSA colonization prevalence of 7.8–38% for residents and 5.2–7.0% for staff members, an ESBL-prevalence of 50–64% for residents and 5.2–14% for staff and a CPE-prevalence of 1.0–6.3% for residents and 0.0–1.5% for staff. These data show that colonization of residents in Italian LTCFs is generally significantly higher than in other European countries. A point prevalence survey undertaken in 2008 in an Italian LTCF [12], repeated in 2012 [13] and 2016 [14], showed high resident colonization rates for ESBL-producing *Enterobacterales* (49.0–64.0%), a low prevalence for CPE (1.7–6.3%) and a variable prevalence for MRSA-colonization (13.2–38.7%). Two recent multicenter studies in Italian LTCFs, confirmed a high colonization prevalence (ranging from 32.8 to 81.5%) for ESBL-producing *Enterobacterales*, especially *Escherichia coli*, whereas lower prevalence was found for CPE (0.0–5.8%) and carbapenemase-producing *A. baumannii* (0.0–5.8%); highly variable prevalence was found for MRSA (5.0–30.6%) and VRE (0.8–20.2%) [15, 16].

An Italian multicenter study, investigating the frequency of ESBL-producing *Enterobacterales* in urine specimens from LTCF residents, found a median ESBL-rate of 32.1%, with *E. coli* as the most frequent ESBL-producing pathogen [17].

In Italian LTCFs, as well as in other European countries, the most prevalent ESBLs from *E. coli* isolates were found CTX-M-type enzymes, particularly CTX-M-15, mainly belonging to the pandemic ST131 clonal group [11, 15, 18]. On the other hand, carbapenemases in *Enterobacterales* from Italian LTCFs, generally are of KPC- or VIM-type [11, 15, 16]. Recently, other authors reported a high colonization rate of residents with KPC-type -producing *Klebsiella pneumoniae* in a LTCF from central Italy: 11.6% of patients were colonized at admission, 9.9% subsequently, with very high rates of carriage and cross-transmission in severe brain injury patients [19].

It is important to emphasize that published surveillance studies from Italian LTCFs are scarce. Moreover, large urban facilities are overrepresented, and there have been no systematic, culture-based or molecular surveys at national or regional level until now.

## Surveillance recommendations for MDROs in Italian LTCFs

Recommended laboratory based surveillance strategies for MDROs range from passive surveillance of routine clinical microbiology laboratory-results (1st level) [20], up to retrospective (2nd level) [2] or prospective surveillance screening (3rd level) [21].

**Table:** 

Surveillance level	Strategies	Organisms included	Recommendation	Limitations
1st level laboratory based surveillance	Surveillance of routine clinical microbiology laboratory-results	Various MDROs	Mandatory	Low number of collected samples, identification of specimens as coming from LTCF patients, exclusion of colonization data
2nd level laboratory based surveillance	Active surveillance cultures	Generally CPE, possible extension to other MDROs	Strongly recommended	Generally limitation to colonization by CPE, exclusion of routine isolates from infections
3rd level laboratory based surveillance	Prospective facility screening of LTCF residents for MDROs	Various MDROs	On a voluntary basis, and recommended	Generally point prevalence study, exclusion of routine isolates from infections, costly and time-consuming

Variation in MDRO surveillance includes facility wide vs. targeted units, patient based vs. laboratory based, colonization vs. infection, passive vs. active, retrospective vs. prospective, and optional vs. required strategies. The significant heterogeneity of Italian LTCFs, as referred to bed number, resident comorbidities and staffing, and the heterogeneity of local, regional and national regulations, complicate the recommendation of generally acceptable surveillance measures for MDROs [7]. Laboratory-based surveillance of MDROs should be associated to a careful infection monitoring -e.g. of incidence of urinary tract infections or central line associated bacteremia-, by healthcare providers within the LTCF, and should also be paralleled by evaluation of the consumption of antibiotics in the institution, as key prerequisites for the implementation of an infection control and antimicrobial stewardship programs [22, 23]. In any case, routine environmental cultures or samples from asymptomatic personnel are not recommended, except as target for an epidemiologic investigation.

## Surveillance of routine clinical microbiology laboratory-results (first level laboratory-based surveillance, mandatory)

We define first level surveillance of MDROs in LTCFs as the cumulative retrospective analysis of antimicrobial susceptibility data, obtained as part of routine care from diagnostic cultures of clinical specimens in the referring clinical microbiology laboratory, in accordance with the Clinical Laboratory Standards Institute (CLSI) guideline for recording and analysis of antimicrobial susceptibility data [5]. Local susceptibility data are mainly used to provide useful information to enable laboratories to assist the clinician in the selection of appropriate therapy for infections, but they can also be used as a basis for infection prevention and control procedures.

Microbiology laboratories chosen by LTCFs for analysis of microbiological specimens must comply with the following criteria:

- accreditation/certification for the analysis of microbiology samples, including implementation of the required internal and external quality control programs
- documentation of an adequate volume of sample processing activity
- capability of cryopreservation of MDR organisms
- ability to collect, elaborate and present cumulative antibiotic susceptibility data

Therefore, specimen processing and data elaboration in a few regional reference laboratories is recommended: it facilitates standardized cumulative retrospective analysis of antimicrobial susceptibility data and their comparison among different LTCFs.

**Fig. 1:**
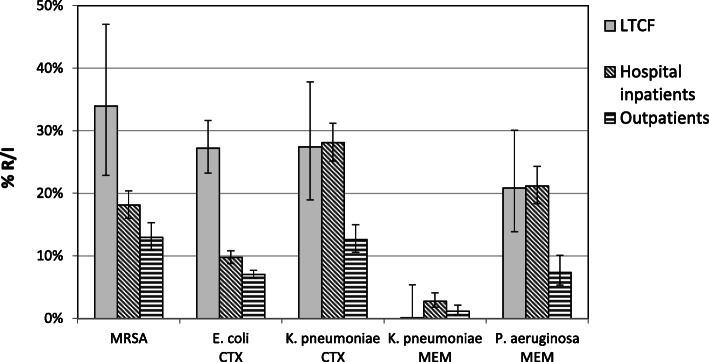
Antibiotic resistance in routine clinical isolates from LTCFs, hospital inpatients and outpatients in the Bolzano healthcare district. Resistance rates were calculated using episode based duplicate exclusion within 28 days (Virtuoso Plus, Dedalus Healthcare Systems Group, Florence, Italy). Screening isolates have been excluded. MRSA: methicillin-resistant *S. aureus*, CTX: cefotaxime, MEM: meropenem. Error bars: 95% confidence interval (https://www.graphpad.com/). Number of isolates: *S. aureus* (LTCFs: 56; inpatients: 1187; outpatients: 918), *E. coli* (LTCFs: 430; inpatients: 3202; outpatients: 6202), *K. pneumoniae* (LTCFs: 84; inpatients: 844; outpatients: 874), *P. aeruginosa* (LTCFs: 96; inpatients: 723; outpatients: 477)

A major disadvantage of using such retrospective susceptibility data is the generally low number of specimens sent to the clinical microbiology laboratory, limiting the number of isolates available for susceptibility testing and cumulative analysis. Therefore, combining of data from consecutive years (e.g. 3 years) and from several comparable LTCFs in a healthcare district area may be required [5]. Elaboration of such combined data is useful for indication of general trends, which can be compared with outpatient or acute-care hospital (ACH) inpatient data from the same geographic region [5]; however, if data are pooled from consecutive years, changes in resistance profiles might be difficult to detect. For example, selective cumulative susceptibility data on routine clinical isolates from LTCFs, inpatients and outpatients from the Bolzano healthcare district, highlighted significantly higher rates of MRSA and cefotaxime-resistance in *E. coli* (mainly ESBL-producers) from LTCF residents, compared with isolates from hospital inpatients; this was not the case for cefotaxime- or meropenem-resistance in *K. pneumoniae* or for meropenem-resistance in *P. aeruginosa* (Fig. 1).

Therefore, an essential requirement for an efficient laboratory-based passive MDROs surveillance system is the mandatory collection of specimens for diagnostic microbiological testing during infectious episodes. Moreover, because of the generally low number of isolates, we recommend to always calculate the 95% confidence interval for antimicrobial resistance rates. Together with the cumulative retrospective analysis of antimicrobial resistance data, a line listing of residents infected/colonized with MDROs should be provided to the infection control practitioners operating in the LTCFs; the line listing can also be prepared for low isolate numbers of single MDRO species (< 30 isolates).

**Fig. 2:**
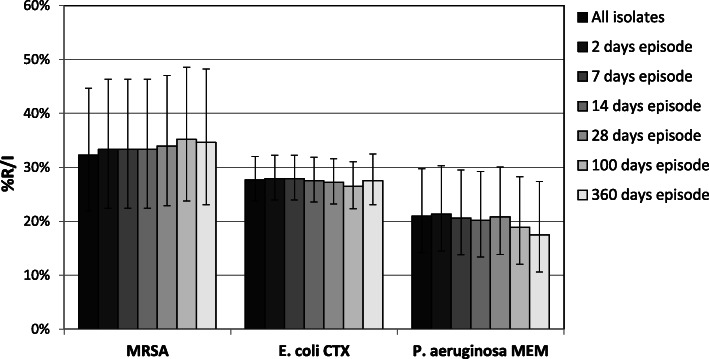
Antibiotic resistance dependent on the method of duplicate isolate removal. Resistance rates were calculated using different methods of duplicate isolate removal (Virtuoso Plus, Dedalus Healthcare Systems Group, Florence, Italy). Screening isolates have been excluded. MRSA: methicillin-resistant *S. aureus*, CTX: cefotaxime, MEM: meropenem. Error bars: 95% confidence interval (https://www.graphpad.com/). Number of isolates (from left to right): *S. aureus* (62–57–57-57-56-54-52), *E. coli* (451–444–444-443-430-393-345), *P. aeruginosa* (105–103–102-99-96-90-80)

Various criteria for exclusion of multiple isolates of the same species from single patients have been proposed [5]. The frequently used first isolate per episode based calculation, includes the first isolate of a given species recovered from each episode of infection, and an episode is defined as the set of all isolates from a patient in which the interval between consecutive isolates is less or equal to a defined time interval, e.g. 28 days. In order to analyze the effect of removal of duplicate isolates by an episode based strategy, we considered all diagnostic isolates (active surveillance isolates excluded) recovered during 2015–17 from LTCF residents in the Bolzano healthcare district, and generated cumulative susceptibility reports for MRSA, cefotaxime-resistant *E. coli* and meropenem-resistant *P. aeruginosa*, based on the all isolates strategy and on different episode-based strategies (minimal interval of time: 2, 7, 14, 28, 100 or 360 days) (Fig. 2). Of note, though a considerable percentage of isolates was removed from calculation in the episode based strategy, with a minimal time interval of 360 days, compared to the all isolates approach (MRSA: 16.1%, *E. coli*: 23.5%, *P. aeruginosa*: 16.4%), the multiple isolates removal did not significantly change resistance rates. Similar studies on the effect of duplicate isolates removal may lead to varying results, depending not only on the method used for the exclusion of multiple isolates, but also on local epidemiology, sampling practice and patient demographics [24]. Recommendations for the application of various episode based duplicate exclusion strategies have been published; it was found that cumulative antimicrobial susceptibility reports based on a 28 days’ episode strategy, reflect resistance rates among hospital-acquired infections [25]. A similar duplicate exclusion strategy has therefore been proposed by an Italian guideline [26]; other authors consider a 10 days episode strategy as first choice for the hospital inpatient setting [24]. LTCF residents stay in the facility for months or years and, due to their underlying chronic diseases, MDROs can give repeatedly rise to infections, requiring frequent antimicrobial treatments. For these reasons, we recommend a 28 days’ episode based strategy for multiple isolates from LTCF residents, but it should be emphasized that the time interval chosen does not significantly change the obtained susceptibility rates; moreover, a 28 days’ episode based exclusion strategy for duplicate isolates has recently been adopted for blood culture isolates by the Italian PNCAR [8].

It is essential that clinical specimens from LTCF residents are clearly associated to a specific LTCF, but this is not always the case in Italian LTCFs; mixing LTCF data with those of ambulatory patients is a major mistake when it comes to tracing the epidemiology of antimicrobial resistance [20].

Patients hospitalized in an LTCF are sometimes transferred to the referral ACH for treatment of serious infections and then they undergo diagnostic cultures of clinical specimens; it is generally a difficult data management problem to include these isolates in the 1st level LTCF surveillance, but results of these cultures should not be missed, as they represent the most serious infectious episodes. A proposed solution to this limitation is to integrate the 1st level LTCF surveillance into the general cumulative antimicrobial susceptibility report, stratifying data according to outpatients, patients hospitalized in the referral ACH and LTCF residents; isolates from residents in LTCFs and from residents admitted to the referral ACH (within 48 h) should, if possible, be pooled together in the drafting of the report.

Based on published international and national guidelines [5, 20, 27], and applying various modifications for LTCFs, for the mandatory first level laboratory-based MDROs surveillance in LTCFs we suggest the following recommendations and strategies.

**Table:** 

Theme of the recommendation	Recommended strategies	Comments	Specific responsibilities
Microbiological specimen collection	Require from LTCF healthcare providers the mandatory collection of specimens for microbiological testing during infectious episodes in LTCF residents	Essential for empiric therapy and for infection control	LTCF physicians, nurses, LTCF infection preventionists
Microbiology laboratory selection	Select laboratories accredited/certified for analysis of microbiological specimens that are also equipped for long-term cryopreservation of MDRO isolates and for the collection, elaboration and presentation of antibiotic susceptibility data	Preferably choose a few regional reference laboratories	LTCF administration, clinical microbiologists
Communication	Communicate immediately the isolation of MDROs from LTCF residents to infection control practitioners in the LTCFs (by phone, fax, e-mail, or smartphone message) and by preliminary reporting	Application of the internal protocol for communication of laboratory-results (e.g. MRSA, VRE, *P. aeruginosa* or *A. baumannii* with MDR phenotype and *Enterobacterales* producing ESBLs, high-level AmpCs and/or carbapenemases)	Clinical microbiologists, LTCF physicians, LTCF infection preventionists
Data elaboration	Produce cumulative antibiograms annually and separately for each LTCF if a sufficient number of isolates per species and year is available (≥30 isolates per species)	If the number of isolates per species and year is < 30, extend the time period up to 3 years and/or include several comparable LTCFs in the same geographic area, in agreement with the infection control practitioners in the involved LTCFs. Prepare for isolate numbers < 30 for single bacterial species a line listing of identified MDROs	Clinical microbiologists and/or epidemiologists
Data elaboration	Exclude isolates from active surveillance and screening studies from the cumulative antibiograms and, if available, elaborate this data separately	Elaborate surveillance and screening data separately (see surveillance levels 2 and 3)	Clinical microbiologists and/or epidemiologists
Data elaboration	Calculate antimicrobial susceptibility rates at species level, focusing on the most important resistance phenotypes	Include at least *S. aureus* (MRSA), *E. faecalis* and *E. faecium* (VRE), *E. coli* and *K. pneumoniae* (ESBL, carbapenemases), *P. aeruginosa* and *A. baumannii* (carbapenem-resistance, carbapenemases)	Clinical microbiologists and/or epidemiologists
Data elaboration	Adhere to the European Committee on Antimicrobial Susceptibility Testing (EUCAST) recommendations to ensure standardized antimicrobial susceptibility testing	Disclose any change in antimicrobial susceptibility testing methodology and interpretative reading (for example therapeutic correction of cephalosporin interpretation for ESBL producers)	Clinical microbiologists and/or epidemiologists
Data elaboration	Use an episode-based strategy for exclusion of multiple isolates per resident, with a minimum default interval of time between their recovery of 28 days	Discuss different other possible removal strategies for duplicate isolates with the LTCF infection control practitioners. Define and disclose the calculation algorithm used for cumulative antimicrobial susceptibility test reports	Clinical microbiologists and/or epidemiologists, LTCF infection control practitioners
Data elaboration	Use stratification of data by specimen type (for example urine, blood, others) only if sufficiently high isolate numbers (≥30 isolates per species and specimen subgroup) have been tested	Discuss various data stratification options with the infection control practitioners in the LTCF	Clinical microbiologists and/or epidemiologists, LTCF infection control practitioners
Data elaboration	Calculate the percentage of MDRO-isolates/total number of isolates × 100 (applying a 28 days’ episode based multiple isolates exclusion strategy)	Always add 95% confidence intervals to susceptibility rates	Clinical microbiologists and/or epidemiologists
Data elaboration	Integrate the LTCF surveillance report into a general cumulative antimicrobial susceptibility data report, comparing isolates from LTCF residents with outpatient isolates and isolates from referral acute care hospital patients	If possible, stratify isolates from LTCF residents admitted within 48 h to the referral ACH together with LTCF isolates	Clinical microbiologists and/or epidemiologists
Data reporting	Report antimicrobial susceptibility rates only for antibiotics routinely tested on all isolates	Do not report supplemental antimicrobial agents that are selectively tested on resistant isolates	Clinical microbiologists and/or epidemiologists
Data reporting	Report antimicrobial susceptibility data as percentages of susceptible-standard dosing regimen, susceptible-increased exposure and resistant isolates	According to EUCAST criteria	Clinical microbiologists and/or epidemiologists
Data reporting	Present the report preferably in a graphic format, easily accessible to the healthcare practitioners in the LTCFs, for example on the institution’s website	Discuss data with healthcare providers in the LTCFs	Clinical microbiologists and/or epidemiologists, healthcare providers in LTCFs
Isolate conservation	Cryopreserve MDR-isolates (at −80 °C)	Preserve for further molecular characterization, in collaboration with a reference molecular biology laboratory	Clinical microbiologists

## Surveillance by active surveillance cultures (second level laboratory-based surveillance, strongly recommended)

Active surveillance of LTCF residents for carriage of MDROs can be defined as obtaining isolates from cultures of specimens that are collected for determining if a patient is harboring a particular organism, and are not from cultures that are obtained as part of the clinical evaluation of a resident’s illness. Active surveillance cultures are specifically designed for MDROs, for which usual reservoirs are established and validated screening tests are available, e.g. CPE [28]. Active surveillance of MDROs can also be based on molecular amplification methods. Use of active surveillance has been shown to improve detection of MDROs, compared with reliance on culture of specimens collected for clinical reasons alone [29].

Specimen types used for active MDRO-screening may influence surveillance data. ESBL- and carbapenemase-producing *Enterobacterales* and VRE are most often recovered from rectal samples (possibly combined with urine samples), whereas we have shown that for MRSA-screening a combination of oropharyngeal (or nasal) and inguinal (or perineal) swabs gives highest yields; testing of only nasal swabs results in substantial underestimation of colonization with MRSA [12–14]. For carbapenem-resistant *P. aeruginosa* and *A. baumannii* oropharyngeal and rectal swabs are recommended (possibly combined with urine samples) [16, 30–32].

According to LTCF infection prevention and control guidelines active surveillance of MDROs in LTCFs by culture methods or by using molecular amplification assays should not imply routine screening of residents at the time of admission to the facility, nor should it be repeated on a periodic basis, in the absence of an epidemic of infections by MDROs, because the application of standard precautions, as applied to all residents, is sufficient [2]. Active surveillance screening for various MDROs should be individualized to residents at risk for shedding large numbers of bacteria into the environment, e.g. residents with colonized wounds not covered fully with dressings, incontinent residents with urinary or fecal carriage, or residents with tracheostomies and difficulty in handling respiratory secretions [1].

CPE, especially KPC-producing *K. pneumoniae*, are epidemically spread in Italy [10, 33] and the emergence of this MDR-phenotype in LTCFs is widely expanding the reservoir of this health-care threat [11, 15, 16, 19]. Based on the current epidemiology and the clinical significance of CPE, we recommend targeting active MDROs surveillance screening in Italian LTCFs on CPE, according to AMCLI indications for the screening of carbapenemases in *Enterobacteriales* [28]; identification of the main carbapenemase types in Italy (KPC, OXA-48-like, VIM, NDM) by antigenic or molecular methods is essential. Though active surveillance in Italian LTCFs is generally restricted to CPE, in accordance with healthcare providers in the LTCFs and taking into consideration the various levels of complexity of the single LTCF, other MDROs may be considered for screening.

GLISTer recommends active routine surveillance screening of MDROs in LTCFs as follows.

**Table:** 

Theme of the recommendation	Recommended strategies	Comments	Specific responsibilities
Selection of MDRO types	Generally restrict active MDRO surveillance screening to CPE	In accordance with healthcare providers in the LTCFs, other MDROs may be considered for screening	LTCF physicians, LTCF infection preventionists, clinical microbiologists
Selection of LTCF residents	Perform active surveillance cultures (or molecular screening) in the presence of infection by CPE (index case), excluding colonization such as asymptomatic bacteriuria	Screening is especially recommended if the index case is at risk for shedding large numbers of bacteria into the environment, e.g. residents with colonized wounds not fully covered with dressings, incontinent residents with urinary or fecal carriage, or residents with tracheostomies and difficulty in handling respiratory secretions	LTCF physicians, LTCF infection preventionists, Clinical microbiologists
Selection of LTCF residents	Perform active CPE surveillance cultures (or molecular methods) on all residents hosted in the same LTCF unit as the index case	Possible extension of surveillance cultures to other close contacts of the index case in other LTCF units; extension of CPE-screening to all residents of the LTCF can be considered, in agreement with the infection control practitioners in the involved LTCFs	LTCF physicians, LTCF infection preventionists, clinical microbiologists
Specimen types	Use rectal or fecal swabs for CPE-screening	Add other specific specimen types for possible screening of other MDROs	LTCF physicians, LTCF infection preventionists, clinical microbiologists
Frequency of surveillance	Repeat CPE surveillance cultures (or molecular screening) as recommended by the infection control professionals of the LTCF	Discuss frequency of possible surveillance for other MDROs with LTCF infection preventionists	LTCF infection preventionists, clinical microbiologists
Environmental or staff screening	Do not perform routine environmental cultures or screening cultures from asymptomatic personnel	Discuss possible extension of screening to environmental samples or asymptomatic personnel (generally not recommended) with infection preventionists, clinical microbiologists, epidemiologists and infectious disease specialists	LTCF infection preventionists, infectious disease specialists, clinical microbiologists, epidemiologists,
Data elaboration	Integrate active MDRO cultural or molecular screening data from LTCF residents into a general antimicrobial susceptibility data report, comparing screening data from LTCF residents with those from referral ACH patients	If possible, stratify screening data from LTCF residents, admitted within 48 h to the referral ACH, together with LTCF screening data	Clinical microbiologists, epidemiologists,
Isolate conservation	Cryopreserve CPE-isolates (at − 80 °C)	Preserve for further molecular characterization, in collaboration with a reference molecular biology laboratory	Clinical microbiologists

## Prospective MDRO surveillance surveys (third level laboratory-based surveillance, on a voluntary basis)

Analysis of routine isolates from clinical diagnostic or active screening cultures obtained from LTCF residents is easy and cheap to perform, but the generally low number of routine clinical samples sent to the microbiology laboratory for analysis limits the value of this strategy; nevertheless, it is generally difficult to include isolates from LTCF residents admitted to the acute care referral hospital in the LTCF surveillance report. Moreover, with respect to the current epidemiological situation of CPE in Italy, the present recommendations for active surveillance screening are focused on CPE. Therefore, prospective surveillance surveys, mainly based on point prevalence colonization studies are recommended, allowing to determine the baseline prevalence of various MDROs in the facility; repeating of the active surveillance can then be used to evaluate the success of an intervention that was implemented in response to high MDRO-rates. We recommend selecting highest prevalence LTCFs for inclusion in the prospective surveillance study, allowing to obtain a “worst case scenario” for a specific healthcare region. Prospective surveillance can be limited to a resident subgroup or may be facility-wide. We recommend performing local, regional and national surveys to delineate the extent of MDRO colonization and infection in residents of LTCFs. For culture based or molecular screening of MDROs, microbiology methods routinely used in the referring microbiology laboratory can be applied. Prospective surveys for MDROs based on culturing specimens from clinically suspected infections can also give significant laboratory-based surveillance results [17].

Molecular typing of the associated isolates is important for identification of resistance plasmids and of hyper-epidemic clonal groups [34], and may be helpful in assessing whether resident-to-resident transmission has occurred. Genotyping can utilize various methods, such as pulsed-field gel electrophoresis (PFGE) [12, 13, 35], polymerase chain reaction and sequence based methods [12, 15, 36], multi locus sequence typing (MLST) [15], up to whole-genome sequencing (WGS) [37], which is rapidly becoming the new standard for the molecular characterization of isolates.

We recommend prospective third level laboratory-based surveillance as follows.

**Table:** 

Theme of the recommendation	Recommended strategies	Comments	Specific responsibilities
Preparation of surveillance project	Prepare a surveillance survey project, in collaboration with the LCTF physicians and the infection control practitioners, to the LTCF administration and the referral ethics committee, that includes institutional review board approval for the survey	Consent from residents (or their legal representatives) to participate is required	LTCF administration, LTCF physicians, LTCF infection preventionists, clinical microbiologists, epidemiologists, local ethics committee
LTCF selection	Select at least one representative LTCF in a healthcare district for performing a point prevalence screening survey, to obtain baseline MDRO colonization data	Selection should be done by the body oversighting the surveillance. Preferably select LTCFs with high prevalence of MDROs (as derived from routine clinical and/or screening data)	LTCF physicians, infection control practitioners, clinical microbiologists, epidemiologists
MDRO types	Include at least ESBL- and carbapenemase-producing *Enterobacterales*, carbapenemase-producing *A. baumannii*, carbapenem-resistant *P. aeruginosa*, MRSA and VRE	Possible extension to other MDROs	Infection control practitioners, clinical microbiologists, epidemiologists
Specimen types	Collect from each LTCF resident at least a rectal (or fecal), an oropharyngeal (or nasal) and inguinal (or perineal) swab	Specimen types recommended for screening of ESBL- and carbapenemase-producing *Enterobacterales* and for screening of VRE are rectal or fecal swabs (possibly combined with urine samples), for carbapenem-resistant *P. aeruginosa* and *A. baumannii* we recommend rectal and oropharyngeal swabs (possibly combined with urine samples), and for MRSA we recommend a combination of oropharyngeal (or nasal) and inguinal (or perineal) swabs (together with swabs from wounds, if present)	Infection control practitioners, clinical microbiologists, epidemiologists
Frequency of surveillance project	Repeat surveillance cultures in the same LTCF at least in a four-year interval	Shorter intervals are preferred	Infection control practitioners, clinical microbiologists, epidemiologists
Staff screening	Perform screening cultures from asymptomatic personnel only if staff members agree to participate in the screening study	Anonymous and only for epidemiologic data collection	Infection control practitioners, LTCF personnel, clinical microbiologists, epidemiologists
Isolate conservation	Cryopreserve MDR-isolates (at −80 °C)	Preserve for further molecular characterization, in collaboration with a reference molecular biology laboratory	Clinical microbiologists

## Infection control measures

Laboratory-based surveillance of MDROs is only effective if the collected data are used by healthcare professionals [38]:

- to better address empiric therapy of infectious diseases
- to guide infection control activities (e.g. isolation precautions for residents to prevent transmission)
- to plan educational programs
- to identify trends in resistance
- to detect outbreaks requiring prompt therapeutic and infection control actions

Exhaustive guidelines providing basic information for infection prevention and control in LTCFs have been published by the Society for Healthcare Epidemiology of America (SHEA) in collaboration with the Association for Professionals in Infection Control (APIC) and by the Royal College of Physicians Clinical Advisory Group on Healthcare Associated Infections [1, 2, 39]. Guidelines agree that for the control of MDROs in LTCFs, besides hygiene measures, the implementation of antimicrobial stewardship programs is pivotal for the improvement of antibiotic use [3].

Finally, any discussion of MDRO control issues in LTCFs must be made in the context of these facilities as a home for residents, in which they usually reside for months or years, and therefore the resident’s living comfort must be addressed together with the control of MDROs. In the absence of risk factors for shedding large numbers of MDROs, LTCF residents should not be restricted from participation in social or therapeutic group activities within the facility, unless there is reason to think that they have been implicated in the development of infections in other residents [2].

## Summary and conclusions

Routine surveillance of MDROs in LTCFs needs to be simple, pragmatic and sustainable. Laboratory-based routine testing of isolates from cultures taken for “clinical” purposes is the most cost-effective and less labor-intensive method to track MDROs. Therefore, we propose this method as mandatory first level laboratory-based MDRO surveillance strategy in Italian LTCFs.

On the other hand, active surveillance cultures (or PCR based methods) report asymptomatic colonization by MDROs (e.g., rectal swabs for CPE). Laboratory-based active screening results may complement routine diagnostic data, and are strongly recommended when an LTCF reports a case of infection by CPE (second level laboratory-based MDRO-screening).

Prospective surveillance surveys for various MDROs (third level laboratory-based MDRO-screening), based on prospective culturing of specimens from clinically suspected infections, and especially on point prevalence studies, is costly and time-consuming, but yields the most significant laboratory-based surveillance results; especially if isolates are molecularly characterized and genotyped.

We therefore recommend a stepwise application of the three MDROs surveillance levels, initially focusing on the elaboration of data from diagnostic bacterial isolates, in a second step extending this procedure to active surveillance screening for CPE, and finally performing prospective point prevalence studies in selected LTCFs. Finally, it is mandatory that the collected and elaborated MDROs surveillance data be used by healthcare professionals to inform decisions for infection prevention and control strategies, targeting the unique aspects of LTCFs.

## Data Availability

Data sharing not applicable to this article as no datasets were generated or analyzed during the current study.

## Abbreviations

GLISTer: Gruppo di Lavoro per lo studio delle Infezioni nelle Residenze Sanitarie Assistite e Strutture Territoriali Assimilabili (Italian working group for the study of infections in LTCFs)
AMCLI: Associazione Microbiologi Clinici Italiani (Italian Association of Clinical Microbiologists)
LTCF: Long-term care facility: encompass nursing homes, residential care centers, chronic disease hospitals, rehabilitation centers and institutions for mentally handicapped persons
MDRO: Multidrug-resistant organism: methicillin-resistant *Staphylococcus aureus* (MRSA), vancomycin-resistant enterococci (VRE), *Pseudomonas aeruginosa* or *Acinetobacter baumannii* with MDR phenotype (resistance to ≥3 classes of antibiotics), *Enterobacterales* producing extended-spectrum β-lactamases (ESBL), high-level cephalosporinases (AmpCs) and/or carbapenemases
PNCAR 2017–20: Piano Nazionale di Contrasto dell’Antimicrobico-Resistenza 2017–20 (Italian plan for contrasting of antimicrobial resistance 2017–20)
